# Acute Myopericarditis with Crohn's Disease Flare-up

**DOI:** 10.7759/cureus.4248

**Published:** 2019-03-13

**Authors:** Manish Kumar, Varun Tandon, Christian M Mosebach, Nerea Lopetegui Lia, Wendy Miller

**Affiliations:** 1 Internal Medicine, University of Connecticut, Farmington, USA

**Keywords:** inflammatory bowel disease (ibd), myocarditis, pericarditis, mesalamine

## Abstract

Cardiac involvement is rare in inflammatory bowel disease (IBD) but can occur as a complication of either the disease itself or drug therapy. We describe an interesting clinical scenario of acute myopericarditis during Crohn’s flare-up.  A 37-year-old patient with severe Crohn's disease started having multiple bloody bowel movements associated with abdominal pain. These symptoms were attributed to Crohn's disease flare-up, prompting the addition of steroids and an increase in the dose of mesalamine without any significant relief. Two weeks later, he presented to the emergency department with pleuritic chest pain. Electrocardiogram (EKG) revealed ST segments elevation in leads I and aVL. Laboratory work revealed elevated troponin I of 1.82 ng/mL, with increased erythrocyte sedimentation rate (ESR) and C-reactive protein (CRP) of 121 mm and 180.1 mg/L, respectively. Cardiac magnetic resonance imaging (MRI) revealed early gadolinium enhancement consistent with myocarditis. The patient was started on colchicine with an increase in the dose of steroids, resulting in clinical improvement. The patient reported having similar chest pain during a previous episode of Crohn's disease flare-up, suggesting underlying IBD as the likely etiology.

## Introduction

Inflammatory bowel disease (IBD) is a systemic disorder predominantly involving the gastrointestinal tract. Extra-intestinal complications are common and frequently affect the skin, musculoskeletal, ocular, and hepatopancreatobiliary systems [1]. Cardiac involvement is rare but can occur as a complication of either the disease itself or medication therapy [2-3]. We present a patient of severe Crohn’s colitis on mesalamine and infliximab, who developed chest pain due to acute myopericarditis in the setting of Crohn’s disease flare-up. 

## Case presentation

A 37-year-old male with a history of severe IBD, on mesalamine and infliximab, and non-ischemic stress-induced cardiomyopathy with a recovered ejection fraction of 50%-55% two years prior presented to the emergency department with complaints of moderate to severe, worsening, constant substernal pleuritic chest pain for three days. He endorsed a dry cough and low-grade fevers. He denied orthopnea, paroxysmal nocturnal dyspnea, weight gain, leg swelling, palpitations, or pre-syncope. He also reported three to four dark, bloody bowel movements a day accompanied by abdominal pain and tenesmus for two weeks. These symptoms were attributed to Crohn's flare-up, prompting the initiation of prednisone 40 mg daily for one week followed by a taper with an increase in the dose of mesalamine from 1200 mg to 1600 mg three times daily. He experienced minimal improvement in his gastrointestinal symptoms. The patient denied the use of tobacco, alcohol, or other illicit drugs. Of note, he reported feeling a similar chest pain during his flare-up two years prior.

**Figure 1:**
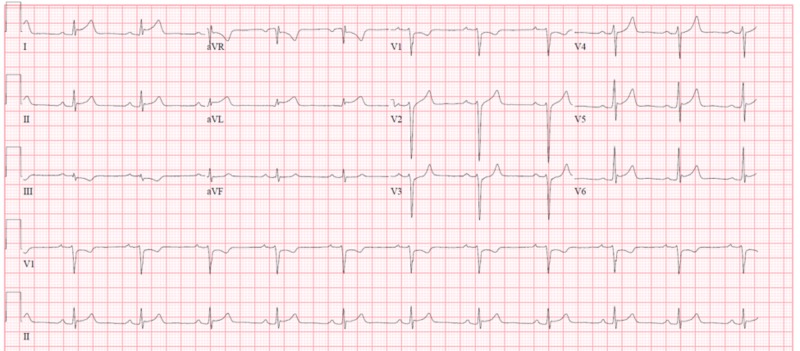
Electrocardiogram (EKG) showing ST segments elevation in leads I and aVL at the time of presentation.

At the time of arrival, the patient was febrile to 100.6^0 ^F, with other vitals unremarkable. Mucous membranes were pale and dry. Lungs were clear to auscultation. The patient had a hyperdynamic precordium, loud S1 and S2, and grade II pan-systolic murmur heard at the apex with radiation to the axilla. He had tenderness to touch in the right lower quadrant with associated guarding and rigidity, but no rebound tenderness. He had regular peripheral pulses with well-perfused extremities. Electrocardiogram (EKG) revealed ST-segment elevation in leads I and aVL (Figure 1). Laboratory work revealed leukocytosis of 26.2 (4.0-10.5 k/uL), chronic stable anemia of 9.5 (12.5-16 g/dL), and unremarkable basic metabolic panel (BMP). Troponin I was elevated to 1.82 (<0.4 ng/mL), ESR 121 (0-22 mm), and CRP 180.1 (<3.0 mg/L). A computed tomography (CT) scan of the abdomen showed distension and wall thickening of the jejunum and proximal sigmoid colon consistent with colitis in the setting of Crohn’s flare-up.

**Figure 2:**
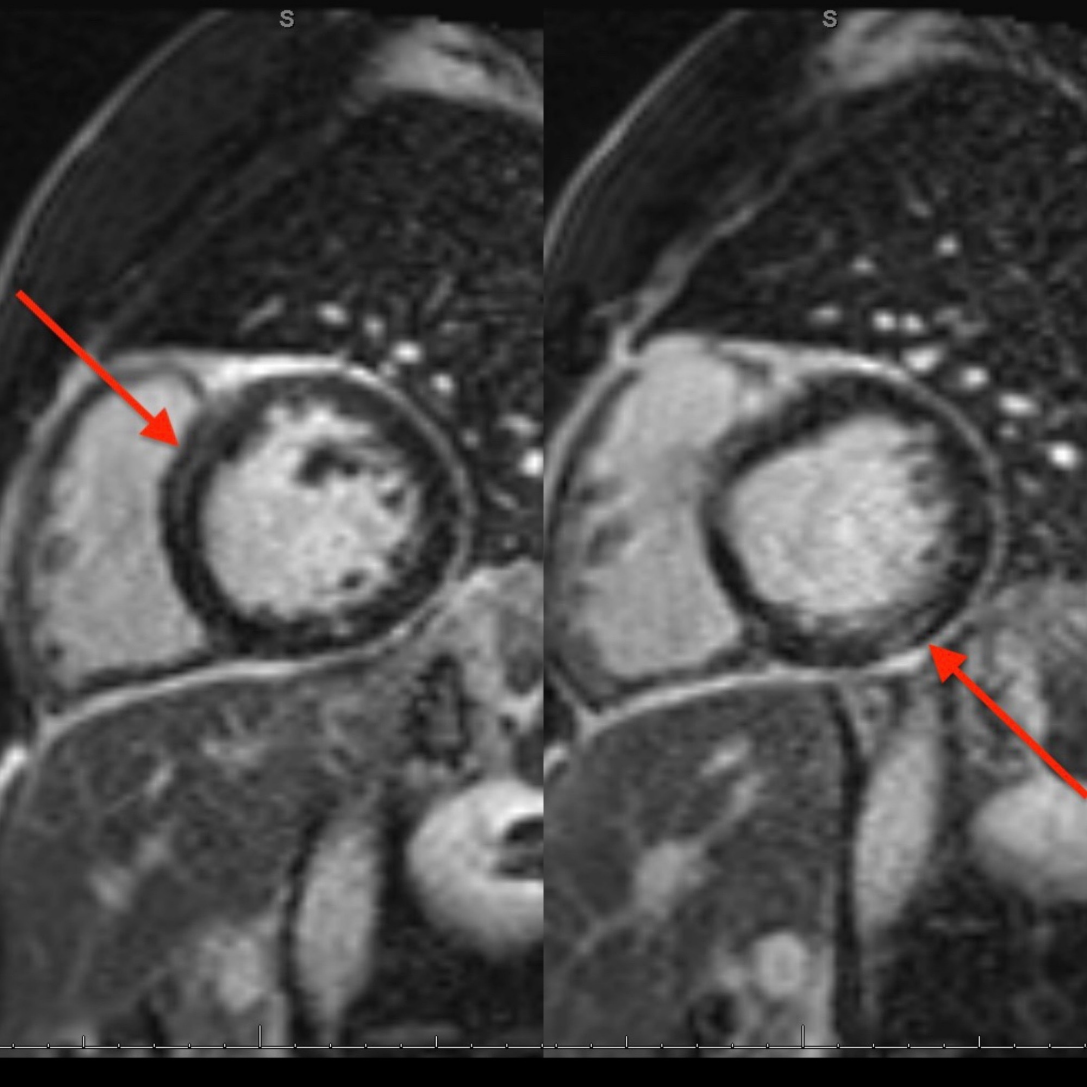
Cardiac magnetic resonance imaging (MRI) showing early gadolinium enhancement (left arrow) and a pattern of patchy mid-myocardial delayed enhancement (right arrow).

ST-segment elevation along with the troponin leak was suspicious for acute coronary syndrome. (ACS). However, two years prior, the patient had an angiogram showing normal coronary arteries. Given the absence of significant atherosclerotic coronary artery disease, his presentation raised suspicion of acute myopericarditis in the setting of Crohn's disease flare-up. The patient was admitted in the hospital and started on colchicine with an increase in the dose of prednisone to 40 mg daily. Mesalamine was discontinued. Cardiac MRI was done and resulted in early and late gadolinium enhancement along with metrics for myocardial edema consistent with myocarditis (Figure 2).

The patient’s symptoms improved with steroids and colchicine. His troponin peaked at 7.21 ng/mL and subsequently trended down. He was eventually discharged on colchicine and a slow steroid taper for 10 days. He followed up with the clinic and was symptom-free.

## Discussion

Inflammatory bowel disease (IBD) is a systemic disorder predominantly involving the gastrointestinal tract. Extraintestinal complications are frequent and mostly affecting the skin, musculoskeletal, ocular, and hepatopancreatobiliary systems [1]. Cardiac involvement is typically rare. Myopericarditis, valvular disease, heart failure symptoms, and thrombosis are the most common cardiac complications [2]. The relative risk of myocarditis is described as 8.3 and 2.6 with Crohn’s disease and ulcerative colitis, respectively, as compared to the general population as per the Danish cohort study [4]. Myopericarditis can occur both due to the underlying disease itself [4-5] or secondary to the drug therapy [3,6-7]. Mesalamine-induced pericardial and myocardial inflammation is well-established [3,6-7] and usually occurs within the first 28 days of initiation of treatment [3]. Also, cases after a long duration of mesalamine exposure have also been described [7]. IBD itself can lead to myopericarditis and may even be the initial presentation of disease [8]. There is no constellation of signs, symptoms, or imaging modalities that can reliably distinguish the etiology of pericardial and myocardial inflammation due to illness or drug therapy. The key distinguishing feature, however, is a temporal association of the signs and symptoms with mesalamine therapy. If the patient has been on mesalamine therapy for a prolonged duration, the occurrence of myopericarditis may very well be due to the underlying disease rather than drugs. The cornerstone of treatment is abrupt discontinuation of mesalamine therapy, typically resulting in a notable resolution of symptoms in a few days [3]. He developed acute myopericarditis in conjunction with Crohn’s flare-up. The dose of mesalamine was increased two weeks before the presentation; however, he had been taking mesalamine for several years without notable adverse cardiac events. He also had reported similar, mild chest pain at the time of the last flare-up of the disease, suggesting that the acute myocarditis was likely due to underlying IBD.

## Conclusions

Myopericardial inflammation is an unusual complication associated with IBD as well as IBD drug therapy. Myocarditis can lead to clinical heart failure and may even be fatal. It is essential to recognize the signs and symptoms of mesalamine-induced cardiomyopathy as early as possible, as the discontinuation of the medication leads to quick resolution of symptoms. Myopericarditis due to IBD should be considered in patients who have been on the 5-ASA agent for an extended period without adverse cardiac effects.
